# Exogenous Vitamin D Mitigates Salt Stress in *Arabidopsis thaliana* via Ion Homeostasis and Calcium‐Mediated Signaling

**DOI:** 10.1002/pld3.70192

**Published:** 2026-10-04

**Authors:** Busra Özdemirci, Okkes Atıcı, Ismail Bezirganoglu, Ozkan Kaya

**Affiliations:** ^1^ Department of Molecular Biology and Genetics Erzurum Technical University Erzurum Turkey; ^2^ Erzurum Technical University High Technology Research Center Erzurum Turkey; ^3^ Department of Biology, Science Faculty Atatürk University Erzurum Turkey; ^4^ Department of Life Sciences Western Caspian University Baku Azerbaijan; ^5^ Republic of Türkiye Ministry of Agriculture and Forestry Erzincan Horticultural Research Institute Erzincan Turkey; ^6^ Department of Horticulture and Agronomy, Faculty of Agriculture Kyrgyz‐Turkish Manas University Bishkek Kyrgyzstan

**Keywords:** antioxidant enzymes, calcium signaling, NaCl toxicity, reactive oxygen species, SOS pathway

## Abstract

Understanding the physiological, biochemical, and molecular responses of *Arabidopsis thaliana* to salt stress and the potential of exogenous vitamin D application to alleviate these responses is critical for developing effective biostimulant strategies under increasing soil salinization. However, knowledge about how vitamin D2 and vitamin D3 mediate salt stress responses at the molecular level remains limited. The physiological, biochemical, and molecular responses of *A. thaliana* Col‐0 seedlings treated with exogenous vitamin D2 (50 ppm) and vitamin D3 (75 ppm) under 100 mM NaCl salt stress were investigated. Treatment significantly influenced all measured parameters. Both vitamin D forms consistently alleviated salt‐induced damage, with combination treatments maintaining higher photosynthetic pigment content, better growth performance, and stronger suppression of oxidative stress markers (reduced MDA, H_2_O_2_, and superoxide anion) compared with salt stress alone. Antioxidant enzyme activities (CAT, POX, APX, GR) were reduced under vitamin D treatment. Ion analysis revealed partial Na^+^ exclusion and elevated Ca^2+^ content under nonsaline vitamin D application, whereas RT‐qPCR analysis demonstrated coordinated upregulation of SOS1 and NHX1 under vitamin D3 combination treatment, alongside modulation of CNGC10, GORK, and SIP3 expression. It is concluded that exogenous vitamin D enhanced salt tolerance in *A. thaliana* through calcium‐mediated activation of Na^+^ exclusion and vacuolar sequestration pathways, with vitamin D3 appearing more effective for growth recovery and ionic regulation at the concentrations tested. The mechanistic insights provided by correlation and clustering analyses suggest that vitamin D operates as a genuine upstream regulator of salt stress signaling rather than a compensatory antioxidant supplement, offering a promising foundation for vitamin D‐based biostimulant applications in saline agriculture. Relative to salt stress alone, the combination treatments lowered MDA by up to about 37% and hydrogen peroxide by roughly 83%, restored chlorophyll a to more than four times the salt‐stressed value, and increased seedling dry weight by approximately 150%.

**Abbreviations**
APX: ascorbate peroxidase
CAT: catalase
CNGC10: cyclic nucleotide‐gated channel 10
EC: electrolyte conductivity
FW: fresh weight
GORK: guard cell outward rectifying K^+^ channel
GR: glutathione reductase
H_2_O_2_
: hydrogen peroxide
HKT1: high‐affinity potassium transporter 1
ICP‐MS: inductively coupled plasma mass spectrometry
MDA: malondialdehyde
MS: Murashige and Skoog
NHX1: vacuolar Na^+^/H^+^ antiporter 1
O_2_•: superoxide anion
POX: peroxidase
ROS: reactive oxygen species
RT‐qPCR: quantitative real‐time polymerase chain reaction
SE: standard error
SIP3: salt‐stress inducible protein 3
SOD: superoxide dismutase
SOS1: salt overly sensitive 1

## 1 Introduction

Salinity is one of the most significant abiotic stress factors limiting plant growth and productivity worldwide (Seyed Hajizadeh et al. 2023). High salt concentrations in the soil impair water uptake by plant roots, creating drought‐like physiological conditions that severely compromise cellular functions. (Awais et al. 2023). Salt stress disrupts essential physiological and biochemical processes, including photosynthesis, respiration, water relations, and ion homeostasis (Muhammad et al. 2024; Dos Santos et al. 2022). The excessive accumulation of toxic ions such as sodium (Na^+^) and chloride (Cl^−^) in plant tissues disturbs ionic balance and generates osmotic stress, which in turn negatively affects plant morphology, leaf water potential, root architecture, dry matter accumulation, and overall yield (Jamshidi Goharrizi, Amirmahani, and Salehi 2020; Jamshidi Goharrizi, Riahi‐Madvar, et al. 2020). It has been estimated that more than 20% of irrigated agricultural lands are affected by salinity, and this proportion continues to rise as a consequence of poor irrigation management and progressive climate change (Fathi et al. 2026; Sasipriya et al. 2026). Furthermore, salt stress not only causes immediate cellular damage but also triggers long‐term epigenetic and transcriptional reprogramming that can alter plant developmental trajectories across generations (Širić et al. 2023; Ibragić et al. 2025). Given the increasing salinization of agricultural lands due to climate change and irrigation practices, understanding plant responses to salt stress and developing strategies to enhance salt tolerance have become critical priorities in plant biology research.

To cope with salinity, plants activate a complex network of physiological, biochemical, and molecular defense mechanisms. At the physiological level, plants accumulate compatible solutes such as proline, soluble sugars, and organic acids to maintain osmotic balance and protect cellular structures from dehydration (Badavath and Saski 2026; Dos Santos et al. 2022). At the biochemical level, the antioxidant defense system plays a key role in mitigating oxidative damage caused by the overproduction of reactive oxygen species (ROS) under salt stress. Enzymatic antioxidants, including superoxide dismutase (SOD), catalase (CAT), peroxidase (POX), ascorbate peroxidase (APX), and glutathione reductase (GR), work in concert to scavenge ROS and protect cellular components from oxidative injury (Jamshidi Goharrizi, Amirmahani, and Salehi 2020; Jamshidi Goharrizi, Riahi‐Madvar, et al. 2020). At the molecular level, salt stress triggers the expression of specific genes involved in ion transport and compartmentalization. The salt overly sensitive (SOS) pathway, particularly the SOS1 gene encoding a plasma membrane Na^+^/H^+^ antiporter, facilitates Na^+^ efflux from cells and reduces ionic toxicity. Similarly, the NHX1 gene encodes a vacuolar Na^+^/H^+^ antiporter that sequesters excess Na^+^ into vacuoles, whereas the HKT1 gene encodes a high‐affinity potassium transporter that regulates Na^+^/K^+^ homeostasis in roots and shoots (Ortega‐Villaizán et al. 2026; Thabet et al. 2026). Furthermore, Ca^2+^ signaling is tightly integrated with salt stress responses, modulating ion transporters and channels that regulate Na^+^ and K^+^ fluxes. In *A. thaliana* , cyclic nucleotide–gated channels such as CNGC10 have been implicated in cation transport, whereas the SIP3 gene is thought to participate in Ca^2+^ metabolism under stress conditions, and the GORK gene encodes a K^+^ channel involved in K^+^ homeostasis (Ma et al. 2026; Seifikalhor et al. 2019; Gong et al. 2021).

Vitamin D refers to a group of lipid‐soluble ketosteroid compounds traditionally associated with calcium homeostasis in animals. However, accumulating evidence over the past decades has demonstrated that both vitamin D2 (ergocalciferol) and vitamin D3 (cholecalciferol) are synthesized in several plant species, primarily through a UV‐B‐dependent pathway involving the conversion of 7‐dehydrocholesterol to cholecalciferol (Jäpelt and Jakobsen 2013). Vitamin D3 and its metabolites have been shown to promote adventitious root formation in *Populus tremula* and *Phaseolus vulgaris* (Buchala and Schmid 1979; Vega et al. 1989), stimulate calmodulin synthesis and Ca^2+^ uptake in bean root segments (Vega et al. 1989), and enhance cell division and DNA synthesis in root meristems through intracellular Ca^2+^ and calmodulin‐mediated pathways (Gong et al. 2021; Niaz et al. 2026). More recently, exogenous gibberellic acid (GA_3_) application has been reported to alleviate salt stress in soybean by promoting plant growth and biomass accumulation under saline conditions (Hamayun et al. 2010). Exogenous application of protective compounds has also been shown to upregulate stress‐responsive gene expression in cucumber (Zhang et al. 2017) and to increase leaf Ca^2+^ content and plant yield in salt‐stressed tomato through the regulation of calcium metabolism (García‐Martí et al. 2019). These findings collectively suggest that vitamin D may play a regulatory role in plant salt tolerance by modulating Ca^2+^ metabolism, yet the detailed mechanisms underlying this protective effect remain largely unexplored, particularly at the molecular level.

Considering the critical roles of antioxidant defense systems, ion transport mechanisms, and Ca^2+^‐mediated signaling in salt stress responses, along with the emerging evidence for vitamin D as a potential modulator of calcium metabolism in plants, this study was designed to comprehensively evaluate the protective effects of exogenous vitamin D application on salt‐stressed *A. thaliana* at the physiological, biochemical, and molecular levels. In this regard, the aim of the present study was to investigate the physiological, biochemical, and molecular effects of applying exogenous vitamin D2 (50 ppm) and vitamin D3 (75 ppm) on *A. thaliana* (Col‐0) seedlings grown under 100 mM NaCl salt stress. Specifically, the following were evaluated: (i) the expression levels of salt stress–responsive genes (SOS1, NHX1, HKT1) and Ca^2+^/K^+^ transport–related genes (SIP3, GORK, CNGC10); (ii) Na, K, and Ca ion contents by inductively coupled plasma mass spectrometry (ICP‐MS); (iii) the enzymatic (SOD, CAT, POX, APX, GR) and nonenzymatic (MDA, H_2_O_2_, O_2_•^−^) antioxidant defense responses; and (iv) growth parameters, photosynthetic pigment contents, proline, total soluble sugar, and total protein levels.

## 2 Materials and Methods

### 2.1 Plant Material and Growth Conditions

*Arabidopsis thaliana* ecotype Columbia‐0 (Col‐0) was used as the model organism in this study. Seeds were obtained from the Department of Biology, Faculty of Science, Atatürk University, Erzurum, Turkey. Seeds were surface‐sterilized with 70% ethanol for 10 min, followed by treatment with 5% sodium hypochlorite for 5 min, and then rinsed three times with sterile distilled water. Sterilized seeds were sown on Petri dishes containing Murashige and Skoog (MS) medium (Murashige and Skoog 1962) supplemented with 0.8% sucrose and 0.8% agar (pH 5.8). Prior to incubation, Petri dishes were maintained at 4°C under continuous light for 4 days to break seed dormancy. Plates were then transferred to a growth chamber and incubated for 21 days under controlled conditions (25°C, 50% relative humidity, 16 h light/8 h dark photoperiod, and 150 μmol photon m^−2^ s^−1^ light intensity).

### 2.2 Preliminary Dose Optimization

To determine the optimal salt concentration, preliminary experiments were conducted using six NaCl concentrations (25, 50, 75, 100, 150, and 200 mM) with three replicates per treatment. No germination or growth was observed at 150 and 200 mM NaCl. The concentration of 100 mM NaCl, which caused approximately 50% growth inhibition relative to the control, was selected for subsequent experiments. For vitamin D dose optimization, four concentrations (10, 25, 50, and 75 ppm) of both vitamin D2 (ergocalciferol) and vitamin D3 (cholecalciferol) were tested under control conditions. Based on germination percentage and seedling growth performance, 50 ppm D2 and 75 ppm D3 were determined as the optimal doses.

### 2.3 Experimental Design

The experiment was conducted in a completely randomized block design with three replicates, each replicate containing 30 seedlings per treatment. Six treatment groups were established: (1) control (MS medium only), (2) 50 ppm vitamin D2, (3) 75 ppm vitamin D3, (4) 100 mM NaCl, (5) 100 mM NaCl + 50 ppm vitamin D2, and (6) 100 mM NaCl + 75 ppm vitamin D3. Seedlings were harvested after 21 days and used for subsequent physiological, biochemical, and molecular analyses. Harvested plant materials were stored at −80°C until further use. Vitamin D2 (ergocalciferol; Sigma‐Aldrich, P9520‐1G; ≥ 98% HPLC) and vitamin D3 (cholecalciferol; Sigma‐Aldrich, C9756‐1G; ≥ 98% HPLC) were used as exogenous vitamin D sources. Stock solutions of vitamin D2 and D3 were prepared separately by dissolving 35 mg of each vitamin in 5 mL of distilled water, corresponding to an approximate stock concentration of 7 mg mL^−1^. A small amount of Tween 20 was added to facilitate dissolution. The vitamin D stock solutions were added to the autoclaved MS medium after sterilization. For 200 mL of MS medium, 1420 μL of vitamin D2 stock solution and 2130 μL of vitamin D3 stock solution were added to obtain final concentrations of 50 and 75 ppm, respectively. Seeds were subsequently sown onto the supplemented MS media. An untreated MS medium without vitamin D or Tween 20 was used as the control, whereas a vehicle control containing Tween 20 but no vitamin D was also included to account for potential effects of the vehicle. Following sowing, the seedlings were grown under controlled conditions in a climate‐controlled growth chamber for 21 days before harvesting.

### 2.4 Growth Parameters

Root and shoot lengths of seedlings were measured, and mean values were calculated for each treatment. For dry weight (DW) determination, plant samples were oven‐dried at 75°C for 48 h and weighed using an analytical balance. Membrane integrity was assessed by the electrolyte conductivity (EC) method as described by Kaya et al. (2013). Root and shoot lengths were measured with a graduated ruler. For the EC assay, fresh leaf samples were immersed in deionized water and the initial conductivity (EC1) was recorded; the samples were then autoclaved to release total electrolytes (EC2), and membrane injury was expressed as the percentage ratio (EC1/EC2) × 100.

### 2.5 Photosynthetic Pigment Content

Chlorophyll a and chlorophyll b contents were determined from fresh leaf tissues according to the method of Lichtenthaler et al. (1983). Absorbance values were measured spectrophotometrically, and pigment concentrations were expressed as milligram per liter. Briefly, fresh leaf tissue was extracted in 80% (v/v) acetone, the homogenate was centrifuged, and the absorbance of the supernatant was recorded at 663 and 645 nm; chlorophyll a and chlorophyll b concentrations were then calculated using the equations of Lichtenthaler et al. (1983): chlorophyll a = 12.21 × A663–2.81 × A645 and chlorophyll b = 20.13 × A645–5.03 × A663 (mg L^−1^).

### 2.6 Proline, Total Soluble Sugar, and Total Protein Contents

Proline content was determined according to the method described by Ábrahám et al. (2010) and expressed as μmol g^−1^ fresh weight (FW). Total soluble sugar content was measured using the method of Qayyum et al. (2011) and expressed as micromole per liter. Total protein content was determined following the method of Sarkar et al. (2020) and expressed as mg g^−1^ FW. Briefly, proline was extracted in sulfosalicylic acid, reacted with acid ninhydrin, and quantified spectrophotometrically at 520 nm against a proline standard curve; total soluble sugar was determined by the phenol–sulfuric acid method with absorbance read at 490 nm; and total protein was measured by the Bradford dye‐binding assay at 595 nm using bovine serum albumin as the standard.

### 2.7 Determination of Oxidative Stress Markers

Lipid peroxidation was assessed by measuring malondialdehyde (MDA) content according to the method of Kaya (2020) and expressed as nanomole per milliliter. Hydrogen peroxide (H_2_O_2_) content was determined following the method of Kaya (2020) and expressed as microgram per gram. Superoxide anion (O_2_•^−^) production rate was measured according to the method of Elstner and Heupel (1976) and expressed as nmol.min^−1^.g^−1^ FW. Briefly, MDA was quantified via the thiobarbituric acid reaction, with absorbance recorded at 532 nm and corrected for nonspecific turbidity at 600 nm; hydrogen peroxide was assayed by the potassium iodide method at 390 nm; and the superoxide anion production rate was determined from the hydroxylamine reaction, with absorbance measured at 530 nm.

### 2.8 Antioxidant Enzyme Activities

For enzyme extraction, fresh leaf tissues (0.5 g) were homogenized in 50 mM potassium phosphate buffer (pH 7.0) and centrifuged. The supernatant was used for the determination of antioxidant enzyme activities. SOD activity was determined according to Elstner and Heupel (1976). CAT activity was assayed following the method of Gong et al. (2008). POX activity was measured as described by Hart et al. (1971). APX activity was determined according to Yamane et al. (2009). GR activity was assayed following the method of Carlberg et al. (1985). All enzyme activities were expressed as EU g^−1^ FW. Briefly, SOD activity was based on the inhibition of nitroblue tetrazolium (NBT) photoreduction at 560 nm, one unit corresponding to 50% inhibition; CAT activity was measured from the decline in H_2_O_2_ absorbance at 240 nm; POX activity was assayed with guaiacol as substrate at 470 nm; APX activity was determined from the decrease in ascorbate absorbance at 290 nm; and GR activity was determined from NADPH oxidation at 340 nm.

### 2.9 Ion Analysis

Sodium (Na), potassium (K), and calcium (Ca) concentrations in plant tissues were determined using ICP‐MS at the Eastern Anatolia High Technology Application and Research Center (DAYTAM), Atatürk University, Erzurum, Türkiye. Harvested seedlings were thoroughly washed with distilled water, oven‐dried at 70°C for 48 h, and ground into a fine powder using a mortar and pestle. The dried plant samples were subjected to acid digestion using a Milestone connect ETHOS UP microwave digestion system. Ultrapure water generated by a Direct‐Q 8 UV ultrapure water system was used during sample preparation and dilution. Following digestion, the samples were appropriately diluted and analyzed using an Agilent 7800 quadrupole ICP‐MS equipped with an SPS‐4 automatic sample preparation/sample introduction system. Sodium, potassium, and calcium were quantified using the calibration procedure of the ICP‐MS system. The measured elemental concentrations were normalized according to the DW of the plant samples and expressed as mg kg^−1^ DW. Quality control included method blanks, calibration verification standards, internal standards, spiked samples, and certified reference materials. Calibration curves for Na, K, and Ca were linear (*R* ^2^ ≥ 0.999), and internal standard, spike, and certified reference material recoveries were within 80%–120%, 90%–110%, and 90%–110%, respectively. Method detection limits were approximately 0.001–0.01 mg L^−1^ for Na and 0.005–0.05 mg L^−1^ for K and Ca, with corresponding quantification limits of approximately 0.003–0.03 and 0.015–0.15 mg L^−1^; duplicate analyses gave relative standard deviations below 5%.

### 2.10 RNA Isolation and Quantitative Real‐Time PCR (RT‐qPCR)

Total RNA was extracted from 0.3 g of plant tissue per treatment using the TRIzol reagent method. Tissues were homogenized in 500 μL TRIzol, incubated at room temperature for 5 min, and 100 μL chloroform was added. After gentle mixing, samples were incubated for 3 min at room temperature and centrifuged at 12,000 rpm for 15 min at 4°C. The aqueous phase (250 μL) was collected, mixed with 250 μL isopropanol, incubated for 10 min at room temperature, and centrifuged at 12,000 rpm for 10 min at 4°C. The RNA pellet was washed with 1 mL of 70% ethanol, centrifuged at 7500 rpm for 5 min at 4°C, and dissolved in 50 μL RNase‐free water. RNA concentration and purity were assessed using an Epoch Microplate Spectrophotometer (BioTek Instruments, Winooski, VT, United States), and samples with A260/A280 ratios between 1.8 and 2.2 were used for downstream applications. First‐strand cDNA was synthesized using a commercial cDNA synthesis kit (Hdrya) containing 5× buffer, dNTPs (10 mM), random primers, reverse transcriptase (200 EU.L^−1^), and 500 ng μL^−1^ RNA template, following the manufacturer's protocol. The thermal cycling conditions for cDNA synthesis were 25°C for 10 min, 50°C for 30 min, 85°C for 5 min, followed by a hold at 4°C. Following cDNA synthesis, 60 μL of autoclaved distilled water was added to minimize primer dimer formation. Expression levels of six target genes (*SOS1*, *NHX1*, *HKT1*, *SIP3*, *GORK*, and *CNGC10*) were analyzed by RT‐qPCR using SYBR Green PCR Master Mix (5× HOT FIREPol EvaGREEN qPCR Supermix). The *GAPDH* gene (AT2G24270) was used as the internal reference gene. The thermal cycling conditions were 95°C for 15 s, 58°C for 20 s, and 72°C for 20 s for 40 cycles. RT‐qPCR was performed on a RotorGene real‐time thermal cycler (Qiagen, Germany). Each experiment was performed in triplicate, and PCR products were verified by electrophoresis on 1.2% (w/v) agarose gel stained with ethidium bromide. The primer sequences used in this study are listed in Table 1. Relative gene expression levels were calculated using the 2^−ΔΔCt^ method (Livak and Schmittgen 2001). In this method, the cycle threshold (Ct) of each target gene is first normalized to that of the reference gene to obtain the ΔCt value; the ΔCt of the control sample is then subtracted from that of each treatment to yield the ΔΔCt value, and the relative expression (fold change) is calculated as 2 raised to the power of −ΔΔCt, so that the calibrator (control) is assigned a value of 1.

**TABLE 1:** Forward and reverse primers used for RT‐qPCR amplification.

Gene ID	Gene	Forward primer (5′ → 3′)	Reverse primer (5′ → 3′)
At5g27150	*NHX1*	ACCGGTCTGATAAGTGCGTATGTTATC	GTTACCCTCAAGCCTTACTAAGATC
At2g01980	*SOS1*	CAAATAAAATGACGACTGTAATCGAC	GTCCTTGCAAATGCAGCATAAAAC
At4g10310	*HKT1*	GATTTGTCCCCACGAATGAGA	CAAAACCAAGAAGCAAGGGAAC
AT4G30960	*SIP3*	GGATACGACGGAGCTAAGGC	GCCACGACTCAAACACACAC
AT5G37500	*GORK*	CGGAATCGTGGGAAGGAGAC	TGGTGGGACACTTCCTTGTG
AT1G01340	*CNGC10*	ACAATTGTGTTGTGTGTATGTGT	ACGGGACCAGTTGCTTGAAT
AT2G24270	*GAPDH*	AGTTGCACTACCAACTGCCTTGCT	AGGTCAACCACGGACACATCAACA

### 2.11 Statistical Analysis

All experiments were performed with three independent biological replicates, and each measurement was conducted in duplicate, with the mean of the two technical readings used as the value for each biological replicate. Data were expressed as mean ± standard error (SE). Statistical analysis was performed using the SPSS 25.0 software (IBM Corp., Armonk, NY, United States). One‐way analysis of variance (ANOVA) was applied, and significant differences among treatment groups were determined by Duncan's multiple range test at *p* ≤ 0.05 significance level. In addition, a two‐way ANOVA was performed to test the main effects of salinity (two levels) and vitamin D (three levels) and their interaction (salinity × vitamin D) on all measured parameters using a general linear model, with effects considered significant at *p* < 0.05. Bar charts and grouped bar graphs were generated using Python 3.11 with the Matplotlib library (Hunter 2007). A colorblind‐friendly palette based on the Wong color scheme (Wong 2010) was applied to all figures to ensure accessibility. Heatmaps illustrating gene expression fold change values were constructed using the Matplotlib library with the veridic colormap. Pearson correlation matrix analysis among physiological, biochemical, and molecular parameters was performed using Python with the Pandas and Seaborn libraries (McKinney 2010; Waskom 2021), and the resulting correlation heatmap was visualized using a diverging color palette. All figures were exported as high‐resolution PNG files (600 dpi) for publication.

## 3 Results

### 3.1 Growth Parameters and Membrane Integrity

Preliminary dose optimization experiments revealed that 100 mM NaCl caused approximately 50% growth inhibition in *A. thaliana* seedlings, whereas no germination was observed at 150 and 200 mM NaCl (Figure 1A). Among the vitamin D concentrations tested (10, 25, 50, and 75 ppm), 50 ppm D2 and 75 ppm D3 promoted the best seedling growth under control conditions (Figure 1B). Root length was significantly affected by the treatments (*p* ≤ 0.05). The longest roots were recorded in the 50 ppm D2 (4.17 cm) and 75 ppm D3 (3.60 cm) groups, both of which were significantly higher than the control (2.73 cm). Salt stress markedly reduced root length to 1.07 cm, and the addition of vitamin D under saline conditions partially restored root growth, with the 100 mM NaCl + 75 ppm D3 treatment yielding 1.60 cm (Figure 2A). Shoot length followed a similar pattern, with the highest value observed in the 75 ppm D3 group (0.766 cm), whereas salt stress alone resulted in the lowest shoot length (0.433 cm). Vitamin D supplementation under salt stress maintained shoot length at levels comparable with the control (Figure 2B). On the other hand, DW was the lowest in the 100 mM NaCl treatment (0.044 g/seedling), whereas the 100 mM NaCl + 75 ppm D3 combination produced the highest DW among all groups (0.110 g/seedling). Under nonsaline conditions, 50 ppm D2 (0.077 g/seedling) also exceeded the control value (0.058 g/seedling) (Figure 2C). The highest EC value was recorded in the 100 mM NaCl treatment (42.7%), whereas the control, 50 ppm D2, and 75 ppm D3 treatments exhibited the lowest values (22.5%–24.0%) (Figure 2D). The combined 100 mM NaCl + 50 ppm D2 and 100 mM NaCl + 75 ppm D3 treatments yielded intermediate EC levels of 33.0% and 30.5%, respectively. Overall, salt stress reduced root length by approximately 61% relative to the control (from 2.73 to 1.07 cm), whereas the 100 mM NaCl + 75 ppm D3 treatment restored it by about 50% relative to salt stress and increased seedling DW by roughly 150% (from 0.044 to 0.110 g/seedling).

**FIGURE 1:**
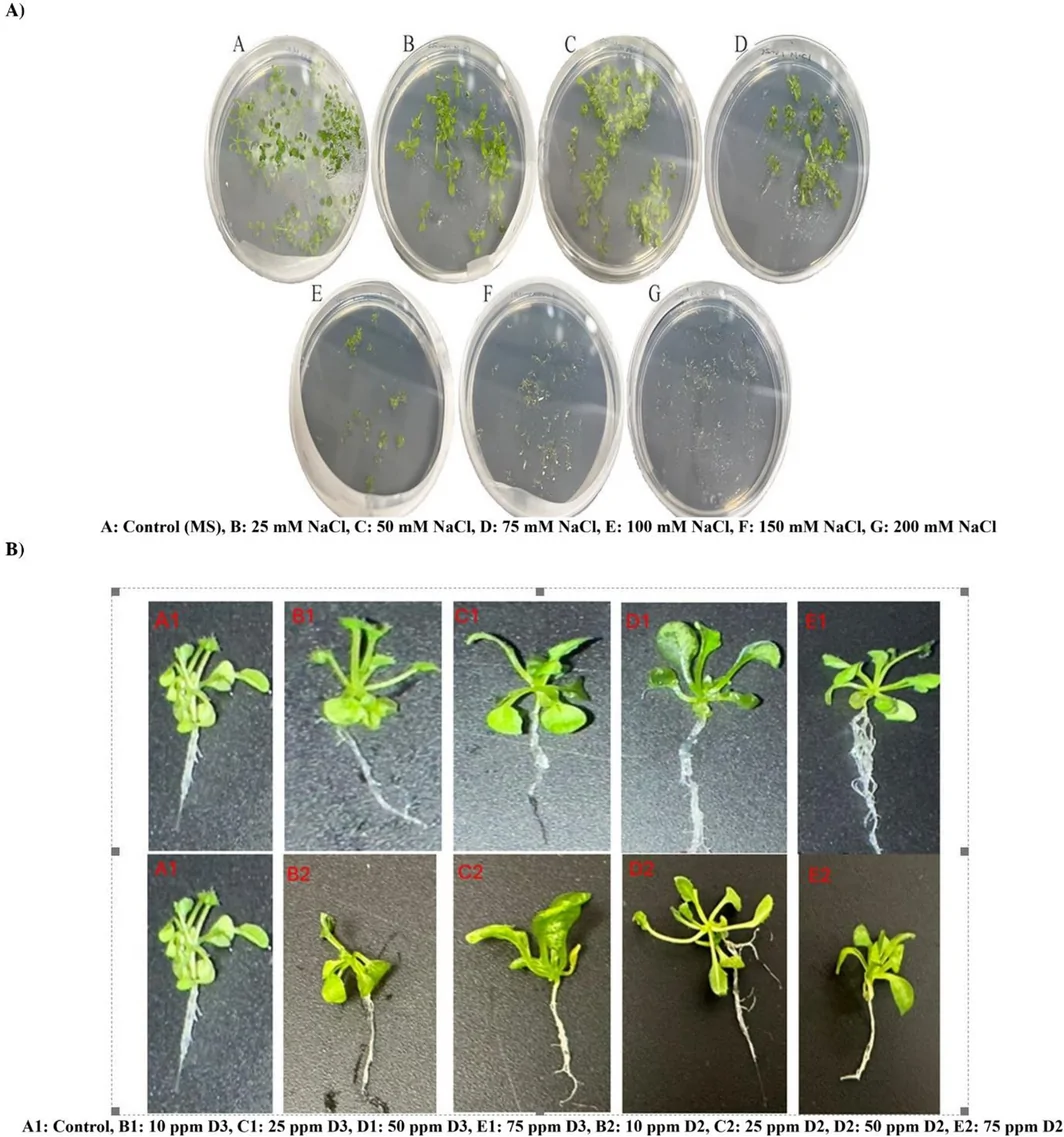
Preliminary dose optimization experiments. (A) Growth of *Arabidopsis thaliana* (Col‐0) seedlings under different salt stress concentrations. No germination or growth was observed at 150 and 200 mM NaCl. The concentration causing 50% inhibition (100 mM NaCl) was selected for subsequent experiments. (B) Growth of *A. thaliana* seedlings under different vitamin D concentrations. Optimal doses were determined as 50 ppm D2 and 75 ppm D3.

**FIGURE 2:**
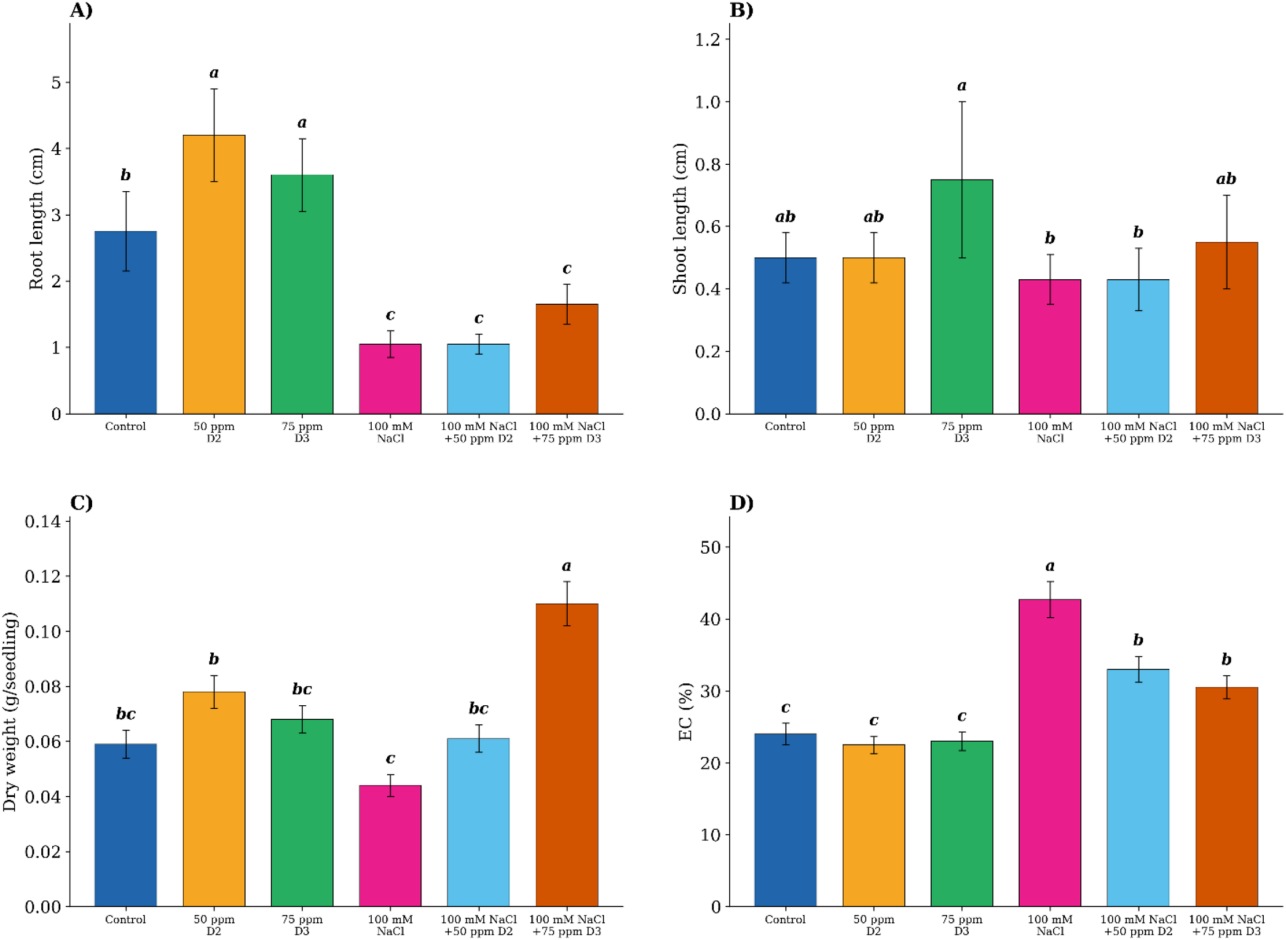
Root length (A), shoot length (B), dry weight (C), and electrolyte conductivity (EC) (D) of *Arabidopsis* seedlings under different treatments. Bars represent mean values ± SE. Different letters indicate statistically significant differences (*p* < 0.05).

### 3.2 Oxidative Stress Markers and Antioxidant Enzyme Activities

Salt stress significantly increased MDA content to 0.326 nmol mL^−1^ compared with the control (0.290 nmol mL^−1^). All vitamin D treatments reduced MDA levels, with the lowest value recorded in the 100 mM NaCl + 50 ppm D2 group (0.206 nmol mL^−1^), which was significantly different from all other treatments (Figure 3A). A similar trend was observed for H_2_O_2_ content, which reached its maximum under salt stress (0.006 μg g^−1^) and was reduced to its lowest levels in the 75 ppm D3 and 100 mM NaCl +50 ppm D2 groups (both 0.001 μg g^−1^) (Figure 3B). Superoxide anion production rate was the highest in the 100 mM NaCl treatment (0.063 nmol g^−1^ FW) and the lowest in the 100 mM NaCl +75 ppm D3 group (0.048 nmol g^−1^ FW FW), which was significantly different from the salt stress group (Figure 3C). Among the antioxidant enzymes, CAT activity increased significantly under salt stress (0.128 EU g^−1^ FW) compared with the control (0.081 EU g^−1^ FW), whereas 50 ppm D2 showed the lowest CAT activity (0.040 EU g^−1^ FW) (Figure 3D). POX activity was markedly elevated under salt stress (1.164 EU g^−1^ FW) and was significantly reduced by vitamin D application, with 50 ppm D2 yielding the lowest value (0.320 EU g^−1^ FW) (Figure 3E). SOD activity did not differ significantly among the treatments (*p* > 0.05), although a numerical increase was observed under salt stress (0.246 EU g^−1^ FW) and a decrease in the 100 mM NaCl + 75 ppm D3 group (0.140 EU g^−1^FW) (Figure 3F). APX activity showed its highest mean value under salt stress (0.097 EU g^−1^ FW) and its lowest in the 100 mM NaCl + 50 ppm D2 treatment (0.038 EU g^−1^ FW) (Figure 3G). GR activity showed a similar numerical pattern, with the highest mean under salt stress (0.083 EU g^−1^ FW) and the lowest means in the 100 mM NaCl + 75 ppm D3 (0.017 EU g^−1^ FW) and 100 mM NaCl + 50 ppm D2 (0.021 EU g^−1^ FW) groups (Figure 3H). For both enzymes, however, within‐treatment variability was high and neither the main effects nor the interaction reached statistical significance in the two‐way ANOVA; these trends should therefore be regarded as indicative rather than conclusive. In quantitative terms, salt stress increased MDA by 12% relative to the control, whereas the 100 mM NaCl + 50 ppm D2 treatment lowered it by roughly 37% relative to salt stress; hydrogen peroxide was reduced by up to about 83% and the superoxide anion production rate by approximately 24% under vitamin D application, and POX activity declined by nearly 73% under 50 ppm D2 relative to salt stress.

**FIGURE 3:**
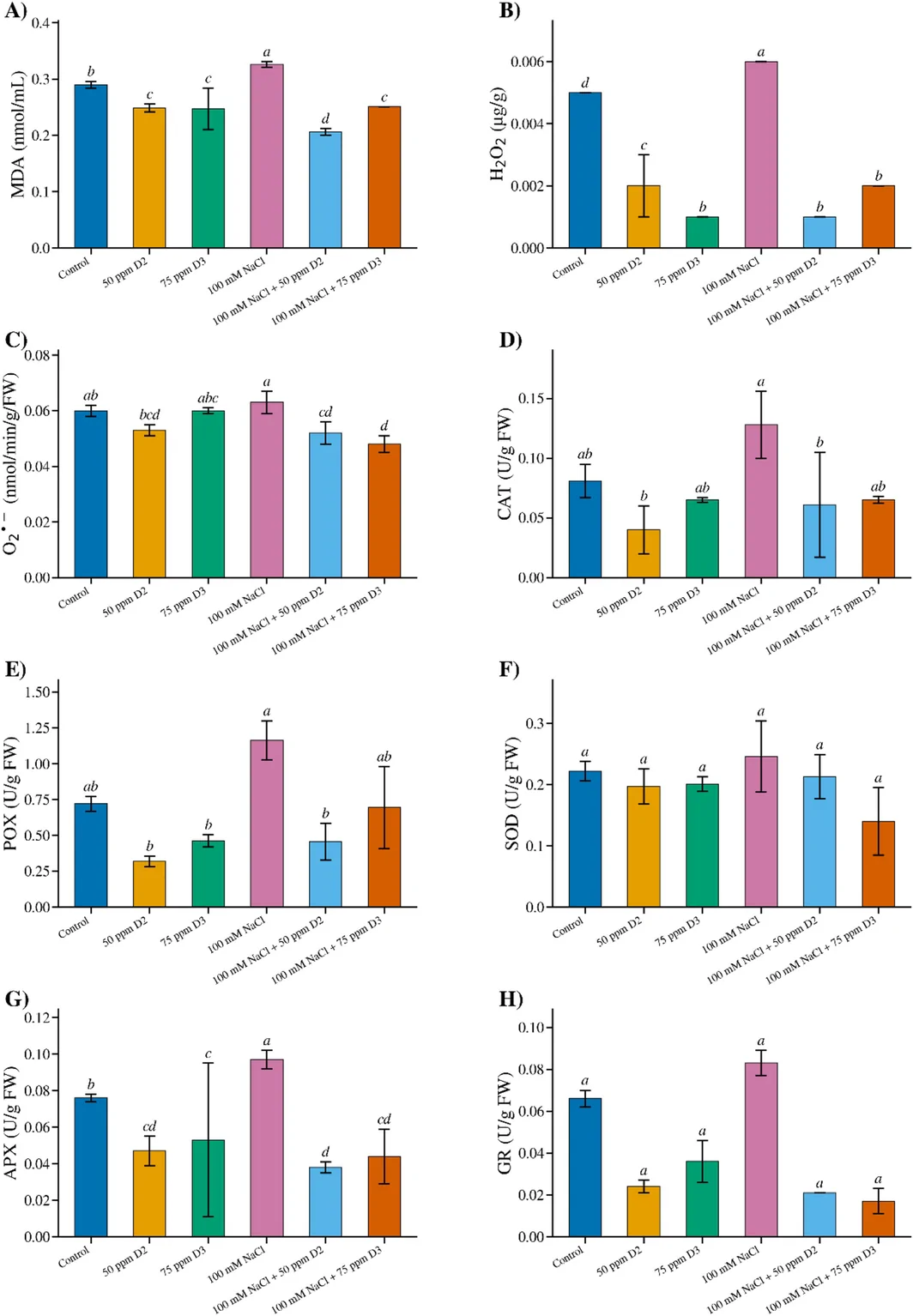
Changes in oxidative stress markers and antioxidant enzyme activities in *Arabidopsis thaliana* under salt stress with vitamin D treatments. (A) MDA, (B) H_2_O_2_, (C) O_2_•^−^, (D) CAT, (E) POX, (F) SOD, (G) APX, and (H) GR. Bars represent mean values ± SE. Different letters indicate statistically significant differences (*p* ≤ 0.05). FW, fresh weight.

### 3.3 Photosynthetic Pigments, Osmoprotectants, and Protein Content

Salt stress caused a sharp decline in chlorophyll a content (0.171 mg L^−1^) compared with the control (0.389 mg L^−1^). The combination treatments markedly increased chlorophyll a level, with the highest values observed in 100 mM NaCl + 50 ppm D2 (0.814 mg L^−1^) and 100 mM NaCl + 75 ppm D3 (0.721 mg L^−1^). Under nonsaline conditions, 75 ppm D3 alone also enhanced chlorophyll a content (0.654 mg. L^−1^). All treatments differed significantly from one another (Figure 4A). Chlorophyll b content exhibited a nearly identical pattern, decreasing to 0.310 mg L^−1^ under salt stress and reaching its peak in the 100 mM NaCl + 50 ppm D2 group (0.874 mg L^−1^) (Figure 4B). Proline accumulation was the highest in the 100 mM NaCl treatment (0.751 μmol g^−1^ FW) and the lowest in the 100 mM NaCl + 50 ppm D2 group (0.477 μmol g^−1^ FW) (Figure 4C). Total soluble sugar content followed a similar trend, with the highest accumulation under salt stress (0.716 μmol mL^−1^) and the lowest in 100 mM NaCl + 75 ppm D3 (0.163 μmol g^−1^), which was significantly lower than the control (Figure 4D). Total protein content was numerically the highest in the 100 mM NaCl group (0.818 mg g^−1^ FW) and the lowest in 100 mM NaCl + 50 ppm D2 (0.625 mg g^−1^ FW); however, no statistically significant differences were detected among treatments (Figure 4E). Quantitatively, salt stress decreased chlorophyll a content by about 56% relative to the control, whereas the 100 mM NaCl + 50 ppm D2 combination raised it more than four‐fold above the salt‐stressed value (from 0.171 to 0.814 mg L^−1^) and roughly doubled it relative to the control; the same combination also reduced proline accumulation by approximately 36% relative to salt stress.

**FIGURE 4:**
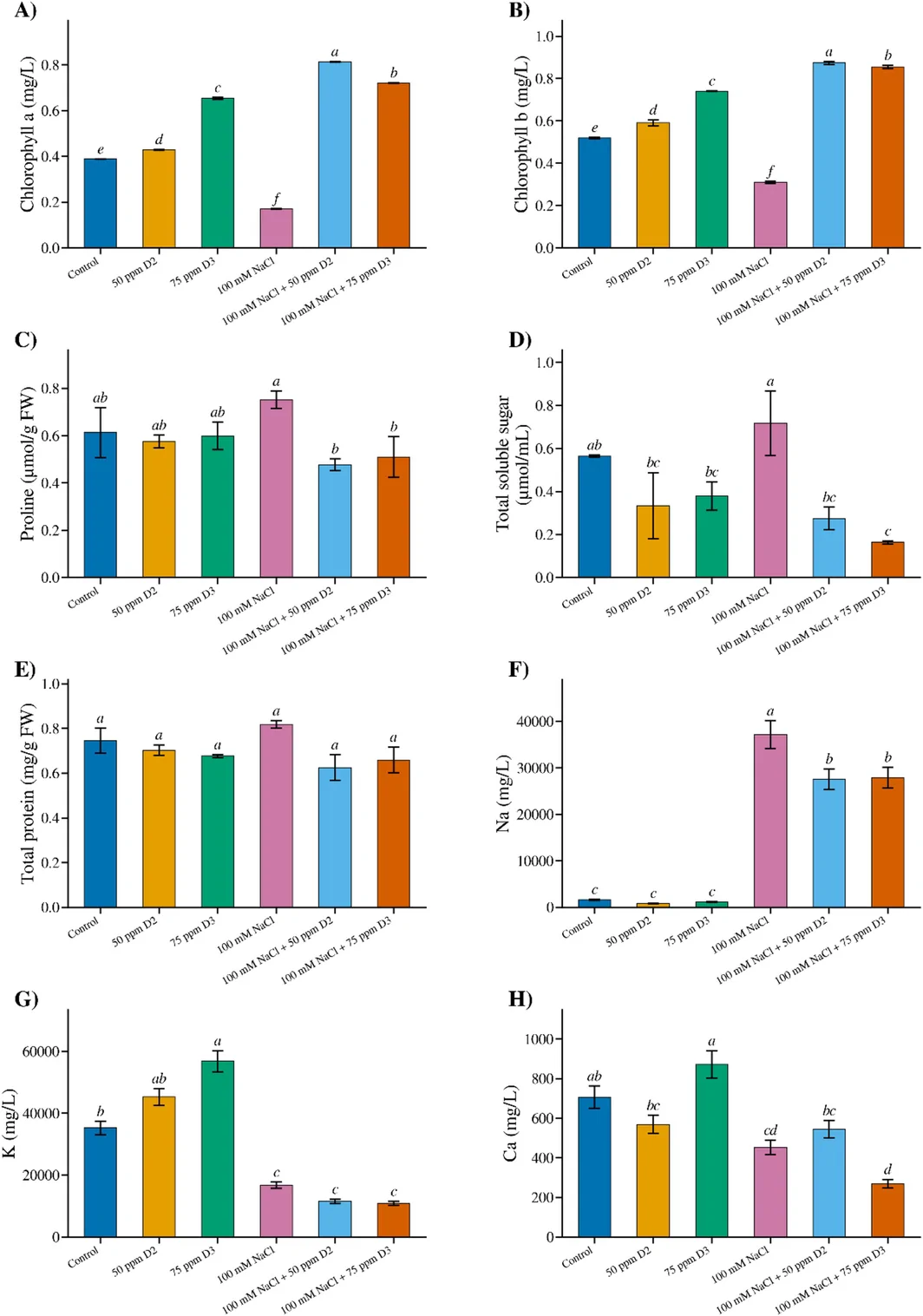
Physiological and ionic parameters of *Arabidopsis thaliana* under salt stress with vitamin D treatments. (A) Chlorophyll a, (B) Chlorophyll b, (C) proline, (D) total soluble sugar, (E) total protein, (F) Na, (G) K, and (H) Ca. Bars represent mean values ± SE. Different letters indicate statistically significant differences (*p* ≤ 0.05). DW, dry weight; FW, fresh weight.

### 3.4 Ion Content and Gene Expression Analysis

All ion contents are expressed on a DW basis (mg kg^−1^ DW). Sodium content increased dramatically under salt stress (37,151.46 mg kg^−1^) compared with the control (1586.39 mg kg^−1^). The combination treatments reduced Na accumulation to 27,550.15 mg kg^−1^ (100 mM NaCl + 50 ppm D2) and 27,902.88 mg kg^−1^ (100 mM NaCl + 75 ppm D3). Under nonsaline conditions, both vitamin D treatments decreased Na content below control levels, with 50 ppm D2 showing the lowest value (807.58 mg kg^−1^) (Figure 4F). Potassium content was the highest in the 75 ppm D3 group (56,819.79 mg kg^−1^) and decreased substantially under salt stress (16,716.87 mg kg^−1^). The combination treatments did not restore K levels, which remained at 11,550.91 mg kg^−1^ (100 mM NaCl + 50 ppm D2) and 10,889.42 mg kg^−1^ (100 mM NaCl + 75 ppm D3) (Figure 4G). Calcium content was the highest in the 75 ppm D3 treatment (871.91 mg kg^−1^) and the lowest in the 100 mM NaCl + 75 ppm D3 group (268.96 mg kg^−1^). Salt stress reduced Ca content to 452.58 mg kg^−1^, whereas 100 mM NaCl + 50 ppm D2 partially maintained Ca levels (544.76 mg kg^−1^) (Figure 4H). In quantitative terms, salt stress increased shoot Na content more than 23‐fold relative to the control (from 1586 to 37,151 mg kg^−1^), whereas both combination treatments lowered Na accumulation by approximately 25% relative to salt stress; potassium and calcium contents under salt stress were reduced by about 71% and 48%, respectively, relative to the 75 ppm D3 treatment.

RNA samples extracted from all treatment groups exhibited A260/A280 ratios ranging from 1.822 to 2.122, confirming acceptable purity for downstream RT‐qPCR analysis (Table 2). Relative expression analysis using the 2^−ΔΔCt^ method revealed distinct patterns among the six target genes (Figure 5). Among the salt stress‐responsive genes, *SOS1* expression was upregulated in the 75 ppm D3 (1.127‐fold), 100 mM NaCl + 75 ppm D3 (1.144‐fold), and 50 ppm D2 (1.008‐fold) groups, while remaining at control level under salt stress alone (1.000‐fold). *NHX1* showed the highest upregulation in the 100 mM NaCl + 75 ppm D3 group (1.252‐fold), followed by 50 ppm D2 (1.178‐fold) and 100 mM NaCl + 50 ppm D2 (1.163‐fold). *HKT1* was upregulated only in the 50 ppm D2 (1.147‐fold) and 75 ppm D3 (1.024‐fold) treatments, whereas salt stress alone and the combination treatments showed expression levels close to the control (Figure 6; Figure 5A,B). Among the Ca^2+^/K^+^ transport‐related genes, *SIP3* was upregulated in the 75 ppm D3 (1.125‐fold), 100 mM NaCl (1.136‐fold), and 100 mM NaCl + 50 ppm D2 (1.061‐fold) groups. *GORK* expression was elevated in the 75 ppm D3 (1.198‐fold) and 50 ppm D2 (1.121‐fold) groups, while remaining near control levels under all salt stress treatments. *CNGC10* exhibited the highest upregulation in the 100 mM NaCl + 50 ppm D2 group (1.379‐fold), followed by 100 mM NaCl + 75 ppm D3 (1.104‐fold) and 100 mM NaCl alone (1.034‐fold) (Figure 5A,B).

**TABLE 2:** RNA concentration and purity of samples from different treatments.

Treatments	260/280 nm	ng/μL
Control	2.085	930.4
50 ppm D2	2.092	1368.4
75 ppm D3	2.085	1182.4
100 mM NaCl	1.822	1122.24
100 mM NaCl + 50 ppm D2	2.122	490.24
100 mM NaCl + 75 ppm D3	2.115	1155.28

**FIGURE 5:**
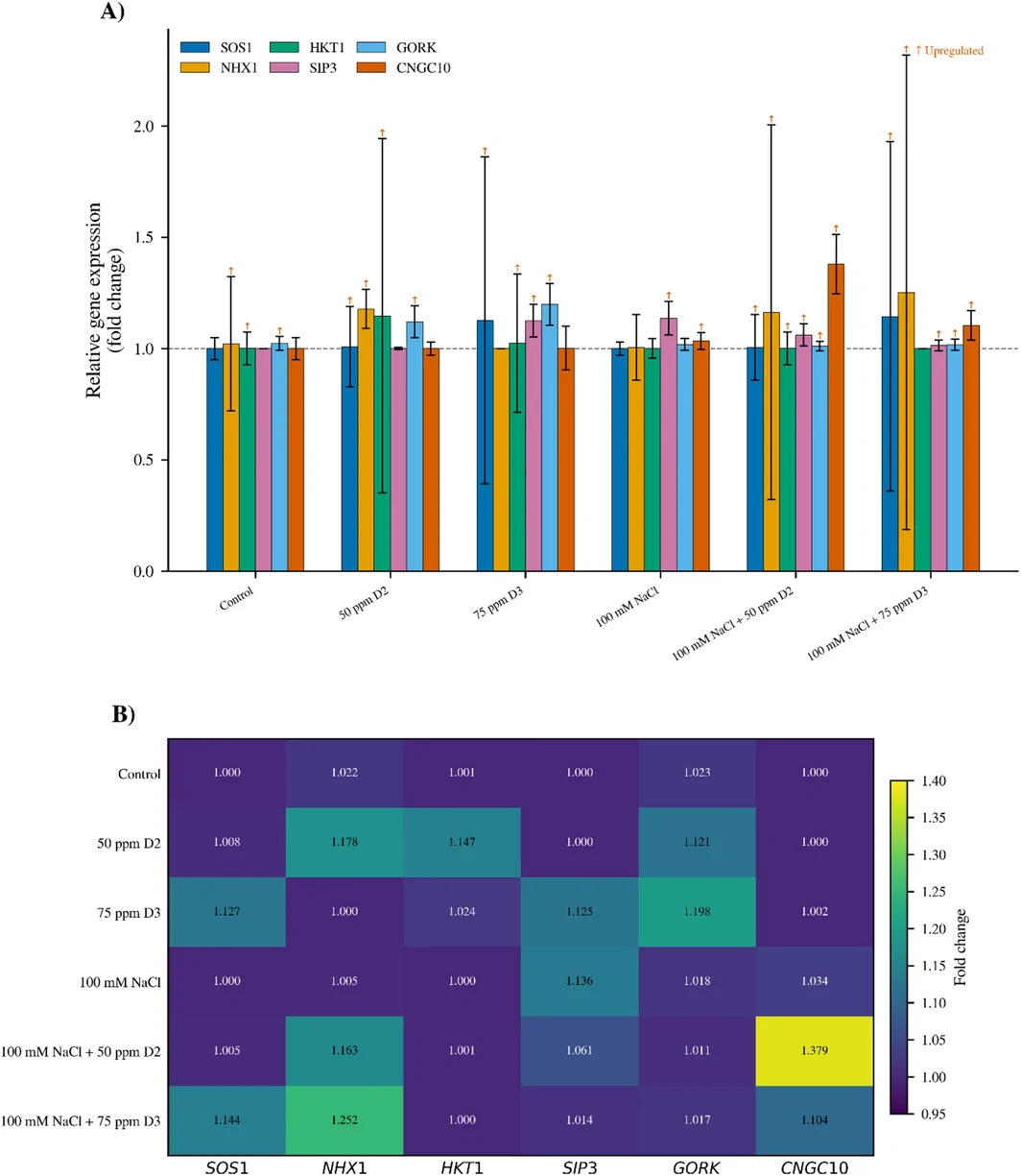
Relative expression levels of salt stress‐related genes (*SOS1*, *NHX1*, *HKT1*, *SIP3*, *GORK*, *CNGC10*) in *Arabidopsis thaliana* seedlings. (A) Bar chart showing mean fold change values ± SE. Dashed line indicates control level (fold change = 1); arrows (↑) denote upregulated genes. (B) Heatmap of gene expression levels based on the 2^−ΔΔCT^ method. Color scale represents fold change intensity.

**FIGURE 6:**
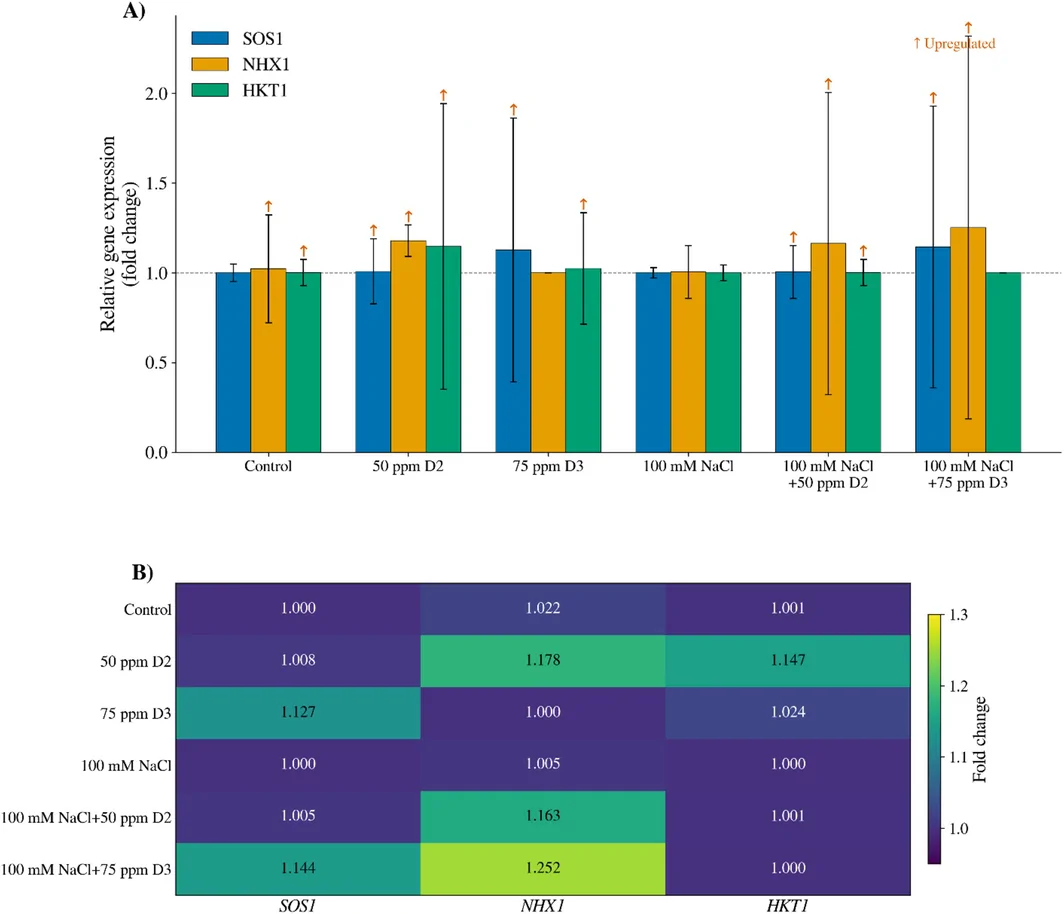
Relative expression levels of salt stress‐related genes (*SOS1*, *NHX1*, *HKT1*) in *Arabidopsis* seedlings. (A) Bar chart showing mean fold change values ± SE. Dashed line indicates control level (fold change = 1); arrows (↑) denote upregulated genes. (B) Heatmap of gene expression levels based on the 2^−ΔΔCT^ method. Color scale represents fold change intensity.

### 3.5 Correlation and Clustering Analysis

Pearson correlation analysis among the physiological and biochemical parameters revealed several notable relationships (Figure 7). Strong positive correlations were observed between shoot length and CAT (*r* = 0.84), proline (*r* = 0.84), protein (*r* = 0.88), and GR (*r* = 0.91). Root length was positively correlated with MDA (*r* = 0.87) and GR (*r* = 0.66). Total chlorophyll content showed negative correlations with CAT (*r* = −0.38), proline (*r* = −0.76), protein (*r* = −0.50), MDA (*r* = −0.73), and superoxide anion (*r* = −0.63), whereas CAT was positively correlated with proline (*r* = 0.78), protein (*r* = 0.88), and APX (*r* = 0.68). Proline exhibited strong positive correlations with protein (*r* = 0.92) and MDA (*r* = 0.79). Among the antioxidant enzymes, GR was negatively correlated with total chlorophyll (*r* = −0.65) and positively correlated with proline (*r* = 0.78). In our results, hierarchical clustering analysis grouped the six treatments into distinct clusters based on their physiological and biochemical profiles (Figure 8). The control and 75 ppm D3 treatments clustered together, indicating similar overall responses. The 100 mM NaCl treatment formed a separate cluster, reflecting its distinct stress‐induced profile. The combination treatments (100 mM NaCl + 50 ppm D2 and 100 mM NaCl + 75 ppm D3) were grouped in close proximity, whereas 50 ppm D2 alone formed an independent branch. The heatmap visualization further illustrated that salt stress was associated with elevated levels of most antioxidant parameters, whereas the combination treatments and vitamin D‐only groups generally exhibited reduced oxidative stress marker levels and enhanced chlorophyll content (Figure 8). On the other hand, the two‐way ANOVA confirmed that vitamin D was the dominant source of variation, with a highly significant main effect (*p* < 0.001) for most parameters (Table 3). More importantly, the salinity × vitamin D interaction was significant for the majority of traits, including root length, chlorophyll a and b and total chlorophyll, MDA, H_2_O_2_, superoxide anion, SOD, CAT, POX, and proline, indicating that the magnitude and direction of the vitamin D response depended on the salinity background. The interaction was nonsignificant for shoot length, soluble sugar, and protein, where the two factors acted independently, and neither factor produced a significant effect on APX or GR activity.

**FIGURE 7:**
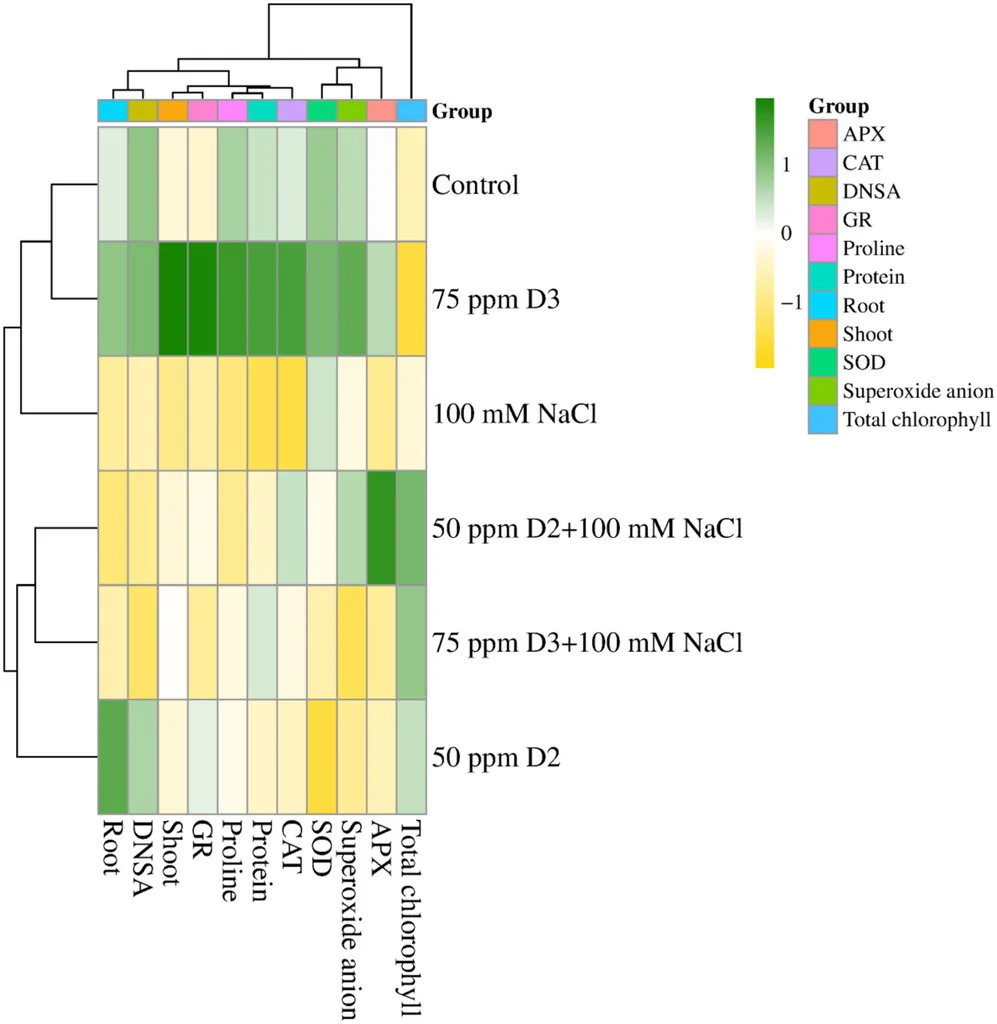
Hierarchical clustering heatmap of physiological and biochemical parameters across six treatment groups in *Arabidopsis thaliana* seedlings. Rows represent measured parameters and columns represent treatment groups: Control, 50 ppm D2, 75 ppm D3, 100 mM NaCl, 100 mM NaCl + 50 ppm D2, and 100 mM NaCl + 75 ppm D3.

**FIGURE 8:**
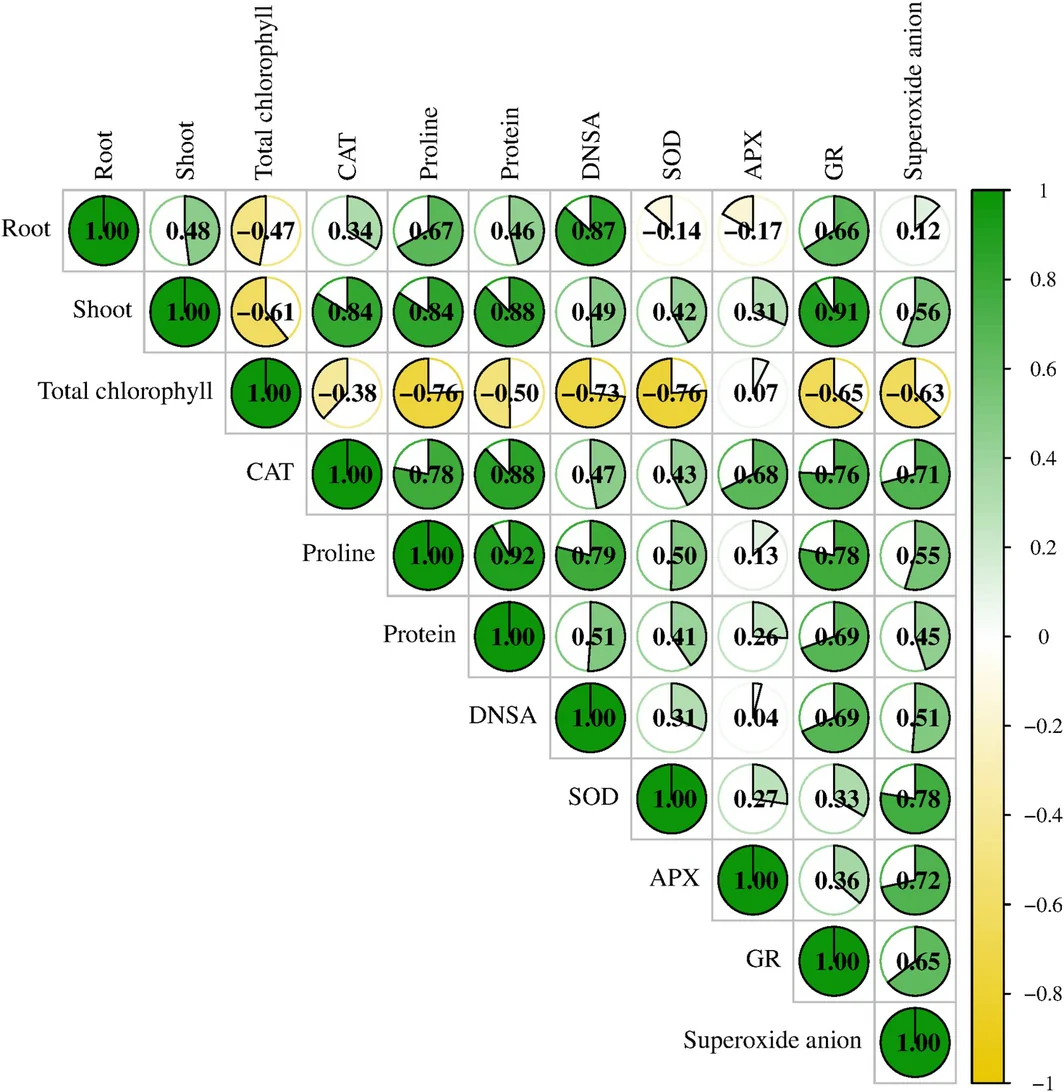
Pearson correlation matrix of physiological, biochemical, and molecular parameters in *Arabidopsis thaliana* seedlings under salt stress and exogenous vitamin D2 and D3 treatments. Color scale represents correlation coefficients ranging from −1 (strong negative, blue) to +1 (strong positive, red).

**TABLE 3:** Two‐way ANOVA (*F*‐values) for the effects of salinity, vitamin D, and their interaction on the measured parameters of *A. thaliana* .

Parameter	Salinity (df = 1)	Vitamin D (df = 2)	Salinity × Vitamin D (df = 2)
Root length	12.9**	42.3***	28.7***
Shoot length	2.9 ns	1.9 ns	1.0 ns
Chlorophyll a	1563.7***	16169.7***	7747.6***
Chlorophyll b	265.2***	3728.5***	1393.7***
Total chlorophyll	1377.3***	16429.2***	6929.9***
MDA	0.1 ns	336.7***	77.1***
H_2_O_2_	0.2 ns	104.7***	4.7*
Superoxide anion	16.6**	167.9***	23.6***
SOD	3.6 ns	135.1***	18.0***
CAT	136.8***	91.4***	27.1***
POX	38.1***	123.1***	12.8**
APX	1.1 ns	0.6 ns	0.8 ns
GR	0.5 ns	1.0 ns	1.4 ns
Proline	9.0*	130.5***	7.6**
Soluble sugar	2.5 ns	6.1*	2.1 ns
Protein	101.0***	139.6***	0.7 ns

*Note:* Error df = 12 for all parameters (*n* = 3 per treatment). Significance:

Abbreviation: ns, not significant.

*** *p* < 0.001.

** *p* < 0.01.

* *p* < 0.05.

## 4 Discussion

### 4.1 Effects of Vitamin D on Growth Parameters, Membrane Integrity, Photosynthetic Pigments, and Osmotic Regulators

Salt stress stands as one of the most damaging abiotic constraints on plant growth and productivity, and the results of our present study confirm this with considerable clarity. The application of 100 mM NaCl reduced root length in *A. thaliana* seedlings by approximately 61% relative to the control, whereas shoot length and DW declined in parallel. These findings are consistent with a large body of literature demonstrating that salt‐induced osmotic and ionic stress impairs cell elongation, cell division, and dry matter accumulation through disrupted water uptake and metabolic interference (Jamshidi Goharrizi, Amirmahani, and Salehi 2020; Jamshidi Goharrizi, Riahi‐Madvar, et al. 2020; Fathi et al. 2026). What is particularly encouraging, however, is the degree to which exogenous vitamin D application partially reversed these growth inhibitions. The 100 mM NaCl + 75 ppm D3 treatment produced the highest DW across all groups (0.110 g/seedling), surpassing even the non‐stressed control (0.058 g/seedling), a result that strongly suggests that vitamin D acts not merely as a stress buffer but as a genuine growth‐promoting agent under hostile conditions. This observation aligns well with earlier reports by Alam et al. (2022), who documented that exogenous vitamin D application improved growth and productivity in salt‐stressed plants, and with Buchala and Schmid (1979), who demonstrated that vitamin D3 metabolites promote adventitious root formation in *Populus tremula* and *Phaseolus vulgaris* . Taken together, these data support the hypothesis that vitamin D operates through an active physiological mechanism in planta, rather than serving merely as a passive biochemical supplement (Raczyk and Carlberg 2026).

The significant salinity × vitamin D interaction detected for most physiological, pigment, and antioxidant parameters (Table 3) provides formal statistical support for the interpretation that vitamin D functions as a salt‐stress modulator rather than a general growth additive: Its effect was conditional on the presence of salt stress rather than constant across backgrounds. The strongest interactions coincided with the pigment and antioxidant traits that also showed the largest treatment contrasts, reinforcing a coordinated, stress‐dependent mode of action. Where the interaction was absent (shoot length, soluble sugar, protein), vitamin D and salinity contributed additively. APX and GR did not respond significantly to either factor, and the numerical trends observed for these two enzymes should be interpreted with caution. Because vitamin D2 and vitamin D3 were applied at different concentrations, the vitamin D factor reflects “treatment” rather than “form” per se, and form‐specific comparisons should still be interpreted with the dose caveat noted above. Chlorophyll a and b contents dropped sharply under 100 mM NaCl stress, chlorophyll a falling to 0.171 mg L^−1^ from 0.389 mg L^−1^ in the control, a result consistent with the well‐documented destructive impact of salt stress on the photosynthetic apparatus, including thylakoid membrane destabilization and chloroplast enzyme inhibition (Gupta and Huang 2014). Short‐term salt stress in *A. thaliana* has been shown to enhance LPO and substantially alter the activities of enzymes involved in protecting photosynthetic membranes (Dos Santos et al. 2022; Niyazi et al. 2026). This pattern is consistent with the elevated MDA and H_2_O_2_ levels recorded under 100 mM NaCl in this study. Remarkably, the combination treatments elevated chlorophyll a far above control levels, with 100 mM NaCl + 50 ppm D2 reaching 0.814 mg L^−1^ and 100 mM NaCl + 75 ppm D3 reaching 0.721 mg L^−1^. A super compensatory increase of this magnitude in stressed *Arabidopsis* has not been widely reported in the vitamin D literature and represents what can be considered one of the mechanistically most significant observations in this study. It is speculated that vitamin D‐mediated modulation of calcium signaling may stabilize thylakoid membranes and upregulate chlorophyll biosynthesis enzymes, though direct experimental testing of this hypothesis is needed.

Proline and total soluble sugar accumulation under salt stress (0.751 μmol g^−1^ FW and 0.716 μmol g^−1^, respectively) reflect the classical osmotic adjustment response of plants under ionic stress, confirming that 100 mM NaCl imposed substantial osmotic imbalance in *A. thaliana* seedlings. Vitamin D application under salt stress dramatically reduced both parameters, the 100 mM NaCl + 50 ppm D2 group yielding the lowest proline and the 100 mM NaCl + 75 ppm D3 group the lowest soluble sugar content. This reduction should not be misread as a weakening of stress tolerance; rather, it most plausibly reflects a diminished need for osmotic compensation because vitamin D application alleviated the underlying ionic and osmotic imbalance, an interpretation supported by the simultaneous improvements in growth and chlorophyll content. Salt stress triggers elevated accumulation of osmolytes such as proline and total soluble sugars as a primary adaptation for osmotic regulation and membrane stabilization, and interventions that genuinely alleviate ionic toxicity characteristically reduce the osmolyte burden (Sharma et al. 2019; Farooq et al. 2025). Our EC results demonstrated that the 100 mM NaCl treatment alone induced the highest EC value (42.7%), reflecting salt‐induced membrane destabilization and increased ion leakage. In contrast, the control and vitamin D‐only treatments maintained the lowest EC levels (22.5%–24.0%), indicating preserved membrane integrity under non‐stressed conditions. The combined 100 mM NaCl + 50 ppm D2 and 100 mM NaCl + 75 ppm D3 treatments yielded intermediate EC values of 33.0% and 30.5%, respectively, suggesting that vitamin D application partially alleviated salt‐induced membrane damage. This interpretation is further supported by the concurrent reductions in MDA and H_2_O_2_ observed in the vitamin D combination groups, indicating that lower EC in these treatments, relative to salt stress alone, reflects genuine membrane protection rather than an artifact. Notably, the correlation matrix revealed strong positive associations between shoot length and both GR (*r* = 0.91) and protein content (*r* = 0.88), collectively suggesting that the vitamin D‐treated seedlings under salt stress maintained a metabolically active, growth‐oriented cellular state rather than a stress‐injured one. The correlation matrix further supports these observations: The strong negative correlations between total chlorophyll and proline (*r* = −0.76), MDA (*r* = −0.73), and superoxide anion (*r* = −0.63) confirm that photosynthetic recovery and oxidative stress burden exist in fundamental tension across treatments, a relationship consistent with the well‐established antagonism between ROS accumulation and chloroplast functionality under ionic stress (Gupta and Huang 2014). Exogenous calcium application in salt‐stressed plants has been shown to simultaneously reduce Na^+^ uptake, enhance proline content, boost antioxidant enzyme activity, and significantly increase photosynthetic pigment content and growth attributes (Islam et al. 2023), a multiparameter pattern that closely mirrors the integrated physiological profile of the vitamin D combination treatments in this study. Hierarchical clustering corroborated this, grouping the control and 75 ppm D3‐only treatments together as physiologically similar, whereas the two combination treatments clustered in close proximity to one another and clearly apart from the salt‐only group, consistent with the view that vitamin D, like calcium, exerts its protective effects through a coordinated, system‐wide mechanism rather than through isolated biochemical adjustments.

### 4.2 Oxidative Stress Markers and Antioxidant Enzyme Activities

Previous studies reported that salt stress‐induced accumulation of ROS represents one of the most universal and well‐characterized aspects of plant responses to salinity (Balasubramaniam et al. 2023; Turfan et al. 2026). In this study, 100 mM NaCl significantly elevated MDA (0.326 nmol mL^−1^), H_2_O_2_ (0.006 μg g^−1^), and superoxide anion production rate (0.063 nmol min^−1^ g^−1^ FW), confirming robust oxidative stress in *A. thaliana* seedlings. Salt stress induces ionic and osmotic stress in plant cells, and a direct consequence of these primary effects is the enhanced accumulation of ROS that are harmful to plant cells at high concentrations; plants have developed complex enzymatic and nonenzymatic scavenging systems to cope with this oxidative challenge (Sardar et al. 2023; Seyed Hajizadeh et al. 2023). All vitamin D treatments, particularly the combination groups, reduced these oxidative markers substantially. The 100 mM NaCl + 50 ppm D2 group produced the lowest MDA (0.206 nmol mL^−1^), and both 75 ppm D3 alone and 100 mM NaCl + 50 ppm D2 reduced H_2_O_2_ to its minimum (0.001 μg g^−1^), demonstrating the potent antioxidant‐modulating capacity of exogenously applied vitamin D. The antioxidant enzyme activities exhibited patterns that, at first glance, may appear paradoxical but become interpretable within the framework of stress severity and protective sufficiency. CAT activity was elevated under salt stress (0.128 U g^−1^ FW versus 0.081 U g^−1^ FW in the control), whereas vitamin D application, particularly 50 ppm D2 alone, brought CAT to its lowest level (0.040 U g^−1^ FW). POX showed a similarly dramatic elevation under salt stress (1.164 U g^−1^ FW) and the sharpest reduction under 50 ppm D2 (0.320 U g^−1^ FW). APX and GR followed analogous trends. APX and GR followed numerically analogous trends, although these differences were not statistically significant (Table 3) and are therefore not interpreted further. Many comparative studies using salt‐tolerant and salt‐sensitive genotypes have correlated salt tolerance with increased antioxidant enzyme activity, yet this relationship is not consistent across all systems; some interventions that genuinely reduce oxidative load produce lower enzyme activities precisely because ROS generation itself has been diminished, removing the inductive stimulus (Sharma et al. 2019; Tripathi et al. 2026). This is precisely the interpretive framework that best fits the present data: Vitamin D application appears to suppress ROS accumulation upstream, thereby reducing the demand on enzymatic scavenging systems rather than directly inhibiting those enzymes. This interpretation is supported by the concurrent and substantial reductions in MDA, H_2_O_2_, and O_2_•^−^ in the vitamin D‐treated groups.

In *A. thaliana* , bacterial inoculation under salt stress increased proline, chlorophyll, and antioxidant enzyme activity while decreasing MDA content, demonstrating that agents capable of alleviating ionic stress tend to produce coordinated improvements across multiple oxidative stress indicators simultaneously (Ahmad et al. 2019; Zhang et al. 2022). The present vitamin D data showed a strikingly parallel pattern. What differentiates the present findings from classical antioxidant supplementation studies is the specificity of the response: D2 and D3 did not uniformly upregulate all enzymes, but rather produced selective modulation consistent with reduced upstream ROS production, with D2 being particularly effective in reducing MDA and H_2_O_2_ levels, and D3 showing comparative advantage in suppressing superoxide anion production; the corresponding changes in APX and GR activities were not statistically significant and are not considered further. This differential efficacy between the two vitamin D forms is itself a noteworthy finding that merits targeted follow‐up study, particularly given that the two forms differ structurally in their side chains, vitamin D2 possessing an additional C22–C23 double bond and methyl group absent in vitamin D3, which may translate into distinct biochemical interactions in planta (Jäpelt and Jakobsen 2013). On the other hand, the Pearson correlation matrix offers additional insight into these biochemical dynamics. The strong positive correlations among CAT, APX, proline, and protein (*r* = 0.68–0.92), along with their collective negative correlation with total chlorophyll (*r* = −0.38 to −0.76), confirm that high‐stress states characterized by active antioxidant enzyme deployment and osmolyte accumulation are fundamentally incompatible with robust photosynthetic function. Vitamin D combination treatments consistently shifted the phenotypic profile away from this high‐stress cluster and toward the chlorophyll‐rich, lower enzyme‐activity cluster, reinforcing our conclusion that these compounds operate primarily by alleviating the ionic and osmotic stress burden rather than by directly potentiating enzymatic defenses. The correlation matrix reinforces this interpretation at the enzymatic level. The strong positive correlations among CAT, APX, proline, and protein (*r* = 0.68–0.92) confirm that high‐enzyme‐activity states are fundamentally associated with high‐stress conditions rather than with stress tolerance per se, a pattern documented broadly across salt stress studies in model plants (Fathi et al. 2026; Turfan et al. 2026). The relative importance of antioxidative defense to overall plant salt tolerance remains a matter of ongoing debate, as some interventions that genuinely reduce oxidative load produce lower enzyme activities precisely because ROS generation itself has been diminished, removing the inductive stimulus for enzyme upregulation (Abogadallah 2010). The consistent separation of the vitamin D combination treatments from the salt‐only cluster in the hierarchical analysis, exhibiting lower enzyme activities together with superior growth and chlorophyll content, supports the conclusion that these compounds reduced upstream ROS generation rather than directly modulating enzymatic capacity.

### 4.3 Ion Content and Salt‐Responsive Gene Expression

The ionic dimension of the salt‐stress response is its most mechanistically complex aspect; together, the present ICP‐MS and RT‐qPCR data provide a coherent, though still incomplete, picture of how vitamin D modulates this dimension in *A. thaliana* . Sodium content increased dramatically under 100 mM NaCl (37,151.46 mg kg^−1^ versus 1586.39 mg kg^−1^ in the control), whereas potassium fell sharply (16,716.87 mg kg^−1^ versus approximately 56,819.79 mg kg^−1^ in the D3‐only group) and calcium was reduced (452.58 mg kg^−1^ versus 871.91 mg kg^−1^ in the D3‐only group). These ion profiling data are fully consistent with the classical ionic toxicity model of salinity, in which Na^+^ influx displaces K^+^ and Ca^2+^ from cellular exchange sites and disrupts the ion homeostasis essential for enzyme function and membrane integrity (Thabet et al. 2026; Niaz et al. 2026; Badavath and Saski 2026). Both combination treatments reduced Na^+^ accumulation (to approximately 27,550–27,903 mg kg^−1^), though they did not restore K^+^ to pre‐stress levels. This partial Na^+^ exclusion is likely connected to the gene expression changes observed. SOS1 was upregulated in the 75 ppm D3 (1.127‐fold) and 100 mM NaCl + 75 ppm D3 (1.144‐fold) groups, consistent with the established role of the SOS pathway in driving Na^+^ efflux across the plasma membrane. SOS1 encodes a plasma membrane Na^+^/H^+^ antiporter whose expression is upregulated in response to NaCl stress, and this upregulation is controlled by the SOS3/SOS2 calcium‐dependent regulatory pathway, making Ca^2+^ availability a central determinant of SOS1‐mediated sodium exclusion (Niaz et al. 2026; Ma et al. 2026). The mechanistically critical implication here is that vitamin D3, by enhancing calcium availability (as indicated by the higher Ca^2+^ content in D3‐treated groups under nonsaline conditions), may potentiate the Ca^2+^‐dependent SOS3–SOS2 signaling cascade that ultimately activates SOS1. This would constitute a genuine upstream regulatory mechanism, rather than a secondary symptomatic response, a distinction that can be considered central to understanding vitamin D's mode of action in plant salt tolerance. However, because the present study relied on gene‐expression and total‐ion measurements rather than direct measurements of cytosolic Ca^2+^ dynamics or transporter activity, this calcium‐mediated mechanism should be regarded as a working hypothesis that requires direct functional validation.

NHX1 was most strongly upregulated in the 100 mM NaCl + 75 ppm D3 group (1.252‐fold), with notable induction also under 50 ppm D2 (1.178‐fold). Salt‐tolerant genotypes consistently show higher expression of NHX1 and SOS pathway‐related genes relative to sensitive genotypes, and the elevation in cytosolic Ca^2+^ concentration has been linked to the expression of Na^+^ efflux ATPases, suggesting a coordinated Ca^2+^‐dependent ionic defense network (Manishankar et al. 2018; Seifikalhor et al. 2019). The concurrent upregulation of both SOS1 and NHX1 under D3 combination treatment is particularly noteworthy: SOS1 drives Na^+^ out of the cell at the plasma membrane, whereas NHX1 sequesters Na^+^ into the vacuole. The simultaneous induction of both genes under the same treatment indicates a coordinated, bi‐directional Na^+^ detoxification strategy that was not fully activated under salt stress alone, and that vitamin D3, plausibly via its calcium‐mobilizing activity, provided the upstream signal necessary to engage both branches of this defense. This, in our view, represents the most important molecular finding of the entire study. HKT1 expression, by contrast, was upregulated only under nonsaline vitamin D treatments (50 ppm D2: 1.147‐fold; 75 ppm D3: 1.024‐fold) and not under combined salt + vitamin D conditions. Higher expression of genes for Na^+^ transport and compartmentation including SOS1, NHX1, and HKT1 in roots contributed to superior salt tolerance in stress‐tolerant grass species, with HKT1 regulating Na^+^/K^+^ homeostasis in a tissue‐specific manner (Liu et al. 2019; Qadir et al. 2026; Sasipriya et al. 2026). The absence of HKT1 induction under saline conditions in the combination groups, despite SOS1 and NHX1 upregulation, may reflect a prioritization of Na^+^ exclusion and vacuolar sequestration over the high‐affinity K^+^ recovery role that HKT1 primarily serves or may reflect the relatively modest K^+^ restoration observed in the ion analysis data.

Among the Ca^2+^/K^+^ transport‐related genes, CNGC10 showed the highest upregulation in the 100 mM NaCl + 50 ppm D2 group (1.379‐fold). CNGC10 negatively regulates salt tolerance in *A. thaliana* by mediating Na^+^ influx, with the cngc10 loss‐of‐function mutant showing greater salt tolerance and lower Na^+^ accumulation in shoots (Hussain 2024; Ortega‐Villaizán et al. 2026). The upregulation of CNGC10 under combined NaCl + D2 treatment therefore warrants careful interpretation: It is possible that elevated CNGC10 expression in this context is a secondary, compensatory signal associated with altered cation flux dynamics rather than a straightforward indicator of Na^+^ loading. GORK expression was elevated specifically in the vitamin D‐only groups (75 ppm D3: 1.198‐fold; 50 ppm D2: 1.121‐fold) but remained near control levels under salt stress treatments. The GORK channel mediates outward K^+^ efflux and is activated by membrane depolarization and ROS, but plants may also use K^+^ efflux as a transient regulatory mechanism to manage initial membrane depolarization caused by salinity until H^+^‐ATPase upregulation restores membrane potential (Shabala 2017; Joshi et al. 2022). The restriction of GORK induction to the nonsaline vitamin D treatments suggests a possible contribution to enhanced root growth in those groups rather than to salt tolerance itself. SIP3 upregulation under 100 mM NaCl and the 100 mM NaCl + 50 ppm D2 combination (1.136‐ and 1.061‐fold, respectively) is consistent with its proposed role in Ca^2+^ metabolism under stress conditions (Gong et al. 2021), and its co‐induction with CNGC10 under the D2 combination treatment hints at a coordinated calcium‐cation signaling response specific to the D2 form. At the molecular level, the clustering analysis assigned the two combination treatments to a closely adjacent branch, distinct from the salt‐only cluster, indicating that both vitamin D forms produced qualitatively similar ionic rescue profiles despite differing in the specific genes most strongly induced. Salt‐tolerant genotypes consistently exhibit higher expression of NHX1 and SOS pathway‐related genes, and elevation of cytosolic Ca^2+^ concentration has been linked to the activation of Na^+^ efflux ATPases, suggesting a coordinated Ca^2+^‐dependent ionic defense network (Seifikalhor et al. 2019). The positive correlation between root length and MDA (*r* = 0.87) warrants careful reading in this context: It reflects cross‐treatment co‐variance rather than a causal relationship, as the D2‐ and D3‐only groups, which had the longest roots, also displayed moderately elevated MDA relative to the severely salt‐stressed group, a metabolic signature of active growth rather than oxidative injury, consistent with the well‐documented role of basal LPO in normal membrane turnover (Jamshidi Goharrizi, Amirmahani, and Salehi 2020; Jamshidi Goharrizi, Riahi‐Madvar, et al. 2020).

## 5 Conclusions

This study revealed that exogenous vitamin D application significantly influences *Arabidopsis thaliana* resilience to salt stress. When vitamin D2 and vitamin D3 were compared, either alone or in combination with NaCl, both forms consistently demonstrated protective capacity across physiological, biochemical, and molecular parameters, with each exhibiting a distinct profile of efficacy. Under combined salt and vitamin D treatment, the seedlings maintained higher photosynthetic activity (elevated chlorophyll a and b), better growth performance (restored root length, shoot length, and DW), stronger suppression of oxidative damage (reduced MDA, H_2_O_2_, and superoxide anion), and more effective ionic regulation (partial Na^+^ exclusion with SOS1 and NHX1 upregulation). The divergent protective profiles between the two forms were particularly evident in their biochemical signatures. Although both forms alleviated salt stress, vitamin D3 showed superior growth‐promoting and Na^+^ exclusion capacity, whereas vitamin D2 showed a comparative advantage in reducing lipid peroxidation and antioxidant enzyme activities. These differences were further reflected in ion homeostasis and gene expression profiles, with the vitamin D3 combination treatment producing the most coordinated activation of both SOS1‐mediated efflux and NHX1‐mediated vacuolar sequestration, pointing toward calcium signaling as the central mechanistic link. These findings have immediate practical implications for developing vitamin D‐based biostimulant strategies in saline agriculture. The superior multiparameter performance of vitamin D3 under salt stress suggests it should be prioritized in future applied studies. Future research should explore the precise calcium‐mediated receptor mechanisms underlying these responses and validate these findings through field trials across economically important crop species. It should be noted that vitamin D2 and vitamin D3 were applied at different concentrations (50 and 75 ppm, respectively); therefore, direct comparisons of their relative efficacy are confounded by dose and should be interpreted with caution.

## Author Contributions

**Busra Özdemirci** and **Ismail Bezirganoglu** contributed to the conceptualization, methodology, and investigation of the study. **Busra Özdemirci**, **Ismail Bezirganoglu**, **Okkes Atıcı**, and **Ozkan Kaya** were involved in data curation, formal analysis, software, visualization, and original draft preparation. Writing, original draft preparation, and writing review and editing were carried out by **Busra Özdemirci**, **Ismail Bezirganoglu**, and **Ozkan Kaya** All authors have read and approved the final version of the manuscript.

## Funding

This study was supported by the Scientific and Technological Research Council of Turkey (TÜBİTAK) under Project No. 123C338.

## Conflicts of Interest

The authors declare no conflicts of interest.

## Data Availability

The data that support the findings of this study are available from the corresponding author upon reasonable request.
